# Occult Hepatitis B Virus Infection among HIV Positive Patients in Nigeria

**DOI:** 10.1155/2014/796121

**Published:** 2014-04-24

**Authors:** Oluyinka Oladele Opaleye, Adeolu Sunday Oluremi, Adetona Babatunde Atiba, Moses Olubusuyi Adewumi, Olatunji Victor Mabayoje, Emmanuel Donbraye, Olusola Ojurongbe, O. Adekunle Olowe

**Affiliations:** ^1^Department of Medical Microbiology and Parasitology, Ladoke Akintola University of Technology, Osogbo, Nigeria; ^2^Department of Virology, College of Medicine, University College Hospital, University of Ibadan, Ibadan, Nigeria; ^3^Department of Hematology, Ladoke Akintola University of Technology, Osogbo, Nigeria; ^4^Department of Medical Microbiology and Parasitology, College of Health Sciences, Obafemi Awolowo University, PMB 4400, Ile-Ife, Nigeria

## Abstract

HIV has been known to interfere with the natural history of hepatitis B virus (HBV) infection. In this study we investigate the prevalence of occult hepatitis B virus infection (OBI) among HIV-infected individuals in Nigeria. Overall, 1200 archived HIV positive samples were screened for detectable HBsAg using rapid technique, in Ikole Ekiti Specialist Hospital. The HBsAg negative samples were tested for HBsAg, anti-HBc, and anti-HCV by ELISA. Polymerase chain reaction was used for HBV DNA amplification and CD4 counts were analyzed by cytometry. Nine hundred and eighty of the HIV samples were HBsAg negative. HBV DNA was detected in 21/188 (11.2%) of patients without detectable HBsAg. CD4 count for the patients ranged from 2 to 2,140 cells/***μ***L of blood (mean = 490 cells/***μ***L of blood). HCV coinfection was detected only in 3/188 (1.6%) of the HIV-infected patients (*P* > 0.05). Twenty-eight (29.2%) of the 96 HIV samples screened were positive for anti-HBc. Averagely the HBV viral load was <50 copies/mL in the OBI samples examined by quantitative PCR. The prevalence of OBI was significantly high among HIV-infected patients. These findings highlight the significance of nucleic acid testing in HBV diagnosis in HIV patients.

## 1. Introduction

Hepatitis B virus (HBV) is a major health problem, with approximately 400 million chronically infected people worldwide, and 15–60% of the normal population in many African countries may be positive for one or more of the serological markers of hepatitis B virus infection [1]. These chronically infected patients not only are at an increased stage of developing liver cirrhosis and hepatocellular carcinoma, but also serve as a potential reservoir of infection [1]. The major structural protein of virus envelope, hepatitis B surface antigen (HBsAg), is universally considered as a diagnostic marker of HBV infection. The absence of HBsAg in the serum and the presence of antibodies to core antigen (anti-HBc) usually indicate resolved infection [2]. Occult HBV infection (OBI) usually has a serological evidence of previous HBV infection that has been described in a few cases [2].

HIV coinfection has been reported to modify the natural history of HBV with potential consequences on morbidity and mortality [3]. Data on OBI in ART untreated HIV patients is limited from a vast number of African countries like Nigeria, where prevalence of HBV monoinfection, mode of transmission, viral genotype, and mutational pattern varies considerably in different parts of the country. No previous study from Nigeria on prevalence on OBI among any groups has been carried out. This is particularly important as exposure to HBV is common among HIV-infected cases because of shared routes of transmission [4]. Notably, there is considerable variation in prevalence of HIV/HBV coinfection according to geographic regions and exposure risk [5].

Successful implementation of ART leads to immune reconstitution that can potentially result in immune mediated liver injury in the setting of HBV coinfection. Some studies have reported an association between OBI and elevated transaminase [6]; therefore identification of OBI is of importance.

## 2. Materials and Methods

Of the 1,200 HIV-infected patients enrolled in the HAART Clinic of the Specialist Hospital, Ikole, Ekiti State, Nigeria, from October, 2012, to April, 2013, we identified 980 HBsAg negative patients (ART-naïve subjects). Among them, 188 were selected for the study by a simple random method. Informed consent was obtained from the patients, and the institutional committee approved the study protocol. The sera were stored at −20°C until tested. Ethical clearance was obtained from the Ethical Committee of the Ikole Ekiti Specialist Hospital.

### 2.1. Serological Testing

All samples were tested for HBsAg, anti-HBs, anti-HBc, anti-HCV, and anti-HIV using ELISA (DRG Diagnostics, Marburg, Germany). All anti-HBc positive samples were retested for HBsAg as well as for anti-HBc, and only repeat positive samples were included in the study.

### 2.2. DNA Extraction

DNA was extracted from all the serum samples using QIAamp DNA Blood Mini kit (Qiagen GmbH, Hilden, Germany) following the manufacturers' instructions. Briefly, samples (200 *μ*L) were incubated with protease and lysis buffer. After incubation, there were two washing steps, and the nucleic acids were eluted in a volume of 50 *μ*L of elution buffer. The eluted DNA was stored at −20°C until tested.

### 2.3. Hepatitis B Virus Specific Nested PCR

The presence of HBV DNA was examined in all samples using a routine diagnostic PCR. Primer pairs were designed from the highly conserved overlapping regions of the S and P genes of the HBV genome. A nested PCR was performed: outer primer pairs were HBPr134 (sense) 5′-TGCTGCTATGCCTCATCTTC-3′ and HBPr135 (antisense) 5′-CAGAGACAAAAGAAAATTGG-3′ and the inner primer pairs were HBPr75 (sense) 5′-CAAGGTTATGTTGCCCGTTTGTCC-3′ and HBPr94 (antisense) 5′-GGTATAAAGGGACTCACGATG-3′. PCR amplifications were carried out in 25 *μ*L reaction volumes with 5 ng of genomic DNA, 10x PCR buffer (20 mM Tris-HCl pH 8.4, 50 mM KCl; Qiagen), 2 mM of dNTPs, 50 ng of each primer, and 1 U Ampli Taq gold DNA polymerase (Applied Biosystems) on a PTC 200 cycler (Peltier Thermal cycler Watertown, Massachusetts, USA). Thermal cycling parameters were initial denaturation at 94°C for 2 min, followed by 35 cycles of 30 sec at 94°C denaturation, 30 sec at 52°C annealing temperature, and 45 sec at 72°C extension, followed by a final extension of 5 min at 72°C. Thermal cycling parameters remained the same as in the first PCR round except for the number of cycles that is increased to 40 cycles of amplification. Each PCR product (5 *μ*L) was analysed by electrophoresis in 2% agarose gels. A positive control (HBV plasmid DNA) and a negative control of the master mix only were integrated to each run to validate the PCR products that yielded a 340 bp fragment.

## 3. Quantification of HBV DNA

Quantification of HBV DNA was performed with quantitative real-time PCR using a previously described procedure [7] in a GeneAmp 7300 sequence analyzer (Applied Biosystems, Perkin-Elmer, Foster City, CA). HBV-plasmid DNA was used to generate a standard curve following a serial 10-fold dilution.

## 4. Statistical Analysis

Mean age and all the numerical data were analysed using Student's  *t*-test. The chi-square test and Fisher's exact test were used to compare categorical data. For the purpose of our study, *P* value ≤0.05 was considered statistically significant.

## 5. Results

The demographic, biochemical, and virological parameters of the study group are summarized in Table 1. The mean age was 35 (range: 3–67) years. The majority (45%) had multiple sexual partners and 25% of the subjects had a history of concomitant alcohol use. HCV coinfection was found in 2/96 (2.1%). Overall, 29/96 (29.2%) of patients were reactive for anti-HBc, an indication of prior exposure to HBV DNA, and majority 6/8 (75%) of the patients were female (Table 2). Thus, in the total study population, 21/188 (11.2%) of patients were identified as OBI and 62.5% of the OBI patients had CD4 count less than 200 cells/mm^3^. Averagely the HBV viral load was <50 copies/mL in the OBI samples examined by quantitative PCR.

**Figure 1:**
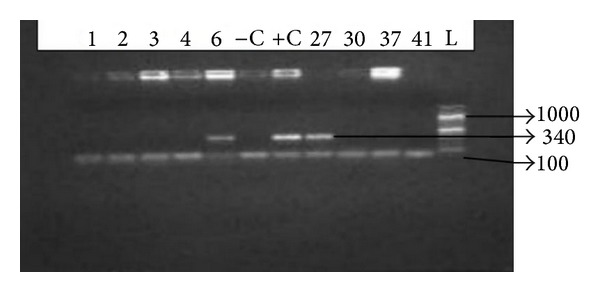
PCR for detection of HBV DNA in HBsAg negative patients. Representative agarose gel electrophoresis of PCR products. Lanes 6 and 27 were positive for HBVDNA. Lanes 1, 2, 3, 4, 37, and 42 were negative for HBVDNA. +C: positive control, −C: negative control, and L: molecular weight size marker.

Serum levels of AST and ALT were higher among patients with OBI in comparison to anti-HBc positive HBV DNA negative individuals, but the difference failed to reach standard significance (*P* = 0.13 and *P* = 0.07), respectively. The comparison of different demographic, biochemical, and virological factors between HBV DNA positive and negative cases was illustrated in Table 3. The distribution of the study participants as per the 1993 Revised Classification System for HIV Infection and Expanded Surveillance Case Definition for AIDS among Adolescents and Adults was as shown in Table 4.  Figure 1 shows the 0.7% agarose gel picture showing a 340 base pairs amplicon.

## 6. Discussion

The present study represents a comprehensive cross-sectional analysis of prevalence of OBI in an ART naïve HIV positive cohort comprising various risk groups. Most previous studies looking at the clinical effects of OBI in HIV include a large number of patients on anti-HBV drugs as a component of ART. This study describes the risk factors associated with OBI, frequently of anti-HBc positivity and its possible values as a serological marker for identifying HIV-infected patients who benefit from HBV DNA assay. We found the prevalence of occult HBV to be 11.2% among a random selected group of HIV-infected patients. The prevalence of OBI in HIV positive individuals varies worldwide between 0 and 90%, depending on the geographic regions, risk factors, and the exposure involved [6].

In the present study, the prevalence of anti-HBc (29.2%; 28 of 96) and OBI (28.6%; 8 of 28) among the ART naïve HIV positive cohort was higher compared to previous report on blood donors from studies done in areas of India, areas which reported 21.3% OBI among the HBsAg negative anti-HBe positive donors [8]. Within Nigeria, HBV and HCV coinfection among HCV-infected patients have been reported sporadically from different regions [9]. Most of the previous studies on HBV/HIV co-infection are aimed at detecting HBV prevalence in the HIV population are based on HBsAg positivity (prevalence 9.9 to 11%), but reports on OBI are scarce. A previous study on intravenous drug users in northeastern India detected a prevalence of 15.9%.

Among the OBI cases, the rate of anti-HBs was lower (2/8; 25%), which may be due to the fact that HIV-infected patients are prone to lose anti-HBs immunity at a higher frequency than the general population [10]. Previous reports suggested that the lower HBV replication was associated with milder hepatic damage [11]. Among the subjects with OBI, elevated ALT or AST was found among 75% (6 of 8) and not significant. However, our study is cross-sectional; therefore evaluation of long term clinical significance of OBI should be better addressed by follow-up studies.

The low level of viral load obtained in this study buttress the findings in another study that showed that showed that almost all OBI cases are infected with replication incompetent HBV, revealing a strong suppression of overall replication activity and gene expression, thereby resulting in a significant reduced viral load [12].

HIV patients are screened for concomitant chronic hepatitis B using HBsAg ELISA, and it is not considered cost-effective to perform HBV DNA testing for all HIV patients in our resource-poor setting. Our study tried to identify possible clinical and serological markers which could guide DNA testing in these patients. OBI is reported to be common among HCV infection, but we found its prevalence to be low among our study group. However, anti-HCV was not tested among the other HIV positive samples attending the Specialist Hospital, Ikole, Ekiti State, Nigeria. Furthermore, none of the risk factors were found to be statistically significant markers of OBI and cannot be used as an independent marker for identifying patients who should benefit from HBV DNA estimation.

However, as one third of the anti-HBc positive negative patients were positive for HBV DNA (8 of 28), it is recommended that HIV positive patients with HBsAg negative/anti-HBc positive patterns should be tested for the presence of HBV DNA irrespective of their anti-HBs status. Nevirapine is commonly included in the first line ART regimens at most treatment centers in Nigeria. Our study identified only 21 subjects with OBI, and all the samples were collected from a single center indicating that results might differ in setting with significant different demographic characteristics. Thus in future multicenter study involving large sample size should be taken up.

## 7. Conclusion

A main implication of the presently viable data is therefore further emphasizing the need for efficient HBV vaccination programs. Overall, the present study highlights the need for screening HBV before the initiation of any HAART containing anti-HBV regimens in HBV/HIV coinfected patients. It necessitates the use of NAT for effective laboratory diagnosis of occult HBV infections in HIV positive patients, especially in developing countries where these assays are not widely available.

## Figures and Tables

**Table 1:** Evaluation of overall demographic, biochemical, and virological parameters of the study populations. *P* values by *t*-test.

Characteristic under analysis	Overall status in the study population	Anti-HBc status in the study population (*n* = 96)		
		Positive *n* = 28 (29.2%)	Negative *n* = 68 (70.8%)	*P* value
Male	48 (50%)	18 (64.3%)	30 (44.1%)	0.05
Female	48 (50%)	10 (35.7%)	38 (55.9%)	0.05
Mean age (range)	35 (3–67)	34 (19–67)	34 (3–55)	0.009
Alcohol addiction	24 (25%)	17 (60.7%)	34 (50%)	0.41
Sexual promiscuity	45 (50%)	11 (39.3%)	14 (20.6%)	0.70
CD4 <200 cells/mm^3^	23 (25%)	13 (46.1%)	14 (20.6%)	0.02
ALT >40 IU/L	27 (28.1%)	12 (42.3%)	15 (22.1%)	0.11
AST >30 IU/L	46 (50%)	17 (60.7%)	34 (50%)	0.14
Anti-HCV positive	2 (2.1%)	1 (3.8%)	1 (1.4%)	0.24
Anti-HBs positive	9 (9.4%)	9 (32.1%)	2 (2.9%)	0.0001

**Table 2:** Evaluation of overall demographic, biochemical, and virological parameters of anti-HBc positive samples. *P* values calculated by Fisher test and *t*-test.

Characteristic	OBI (anti-HBcAg +ve) (*n* 8/28)		
	HBV DNA +ve (*n* = 8) Anti-HBc +ve	HBV DNA –ve (*n* = 20) Anti-HBc +ve	*P* value
Male	2 (25%)	6 (30%)	0.47
Female	6 (75%)	14 (70%)	0.47
Mean age (range)	34.5 (12–67)	34 (3–55)	0.48
Alcohol addiction	3 (34.5%)	6 (30%)	1.00
Sexual promiscuity	3 (37.5%)	13 (65%)	0.15
CD4 <200 cells/mm^3^	5 (62.5%)	7 (23.3%)	0.47
ALT >40 IU/L	6 (75%)	8 (26.7%)	0.13
AST >30 IU/L	7 (87.5%)	12 (60%)	0.07
Anti-HCV positive	1 (1.3%)	1 (5%)	0.53

**Table 3:** Comparison of different demographic, biochemical, and virological factors between HBV DNA positive and HBV DNA negative.

Characteristics	HBV DNA negative (*n* = 167)	HBV DNA positive (*n* = 27)
Male	48 (28.7%)	7 (26%)
Female	119 (71.3%)	14 (51.2%)
Mean age (range)	35 (12–67)	34 (3–55)
Alcohol addiction	41 (24.6%)	11 (40.7%)
Sexual promiscuity	50 (30%)	21 (77.8%)
CD4 (mean)	410	215
ALT >40 IU/L	41.2%	58%
AST >30 IU/L	39.8%	72.6%

**Table 4:** The distribution of the Human Immunodeficiency Virus (HIV) infected in study participants as per Centers for Disease Control classification for HIV-infected adults and adolescents with the mean CD4 lymphocyte count in each category (WHO, 2009).

Category		No of patients	Mean CD_4_ count (per mm^3^)
1	T cells >500 cells/mm^3^	69	506
2	T cell 200–499 cells/mm^3^	81	354
3	T cells <200 cells/mm^3^	38	143
